# Editorial: Implementing digital twins in healthcare: pathways to person-centric solutions

**DOI:** 10.3389/fdgth.2025.1741466

**Published:** 2025-12-02

**Authors:** Antonella Carbonaro, Alberto Marfoglia, Luigi Quaranta, Sabato Mellone, Filippo Lanubile

**Affiliations:** 1 Department of Computer Science and Engineering, University of Bologna, Bologna, Italy; 2 Department of Computer Science, University of Bari, Bari, Italy; 3 Department of Electrical, Electronic and Information Engineering “Guglielmo Marconi”, University of Bologna, Bologna, Italy

**Keywords:** digital twin, personalized medicine, 4P medicine, patient-centric care, artificial intelligence in medicine

## Abstract

Over the past decade, digital twins (DTs) have evolved from an engineering metaphor into a powerful paradigm for healthcare innovation. By dynamically linking physical and digital representations of patients, devices, and clinical processes, DTs enable continuous learning systems where data, knowledge, and decision-making converge. This transformation goes far beyond simulation: it redefines how we understand, monitor, and personalize health, moving toward predictive, preventive, personalized, and participatory (4P) medicine. The Research Topic “*Implementing Digital Twins in Healthcare: Pathways to Person-Centric Solutions*” brings together 8 multidisciplinary contributions that explore the translation of digital twin concepts into practical, ethical, and sustainable healthcare applications. Collectively, the works emphasize that DT implementation is not a purely technological endeavor, but rather a systemic, epistemological, and human-centered transformation of care.

## 1 Topic overview

Digital Twins (DTs) are redefining the boundaries of healthcare innovation, offering dynamic, data-driven models that mirror patients, organs, or entire health systems. Originating from industrial engineering, DTs are now being reimagined as intelligent infrastructures capable of real-time simulation, prediction, and personalization. In healthcare, their implementation promises to enable predictive, preventive, personalized, and participatory (4P) medicine. However, translating these promises into practice entails complex sociotechnical challenges, spanning data governance, interoperability, ethical alignment, and user trust.

This Research Topic, “*Implementing Digital Twins in Healthcare: Pathways to Person-Centric Solutions*,” gathers contributions from interdisciplinary teams exploring how DTs can move from conceptual prototypes to person-centered, ethically grounded, and interoperable healthcare solutions. The eight papers published in this Research Topic collectively demonstrate that digital twins are not only technical constructs but evolving socio-technical systems that connect people, data, and decisions.

## 2 Contributions to the Research Topic

In “*Digital Twin for Personalized Medicine Development*,” Saratkar et al. propose a multi-layered DT architecture integrating AI, IoT, blockchain, and extended reality to build living, adaptive representations of patients. Their framework redefines healthcare analytics as a continuous learning process, where predictive modeling and real-world feedback converge to optimize interventions at both individual and population levels.

Haykal's perspective, “*Digital Twins in Dermatology: A New Era of Personalized Skin Care*”, extends the DT paradigm to dermatology through the concept of a skin twin. By integrating genetic, environmental, and behavioral data, this model exemplifies how DTs can anticipate disease onset and empower patients to co-manage their health. Haykal emphasizes that person-centricity requires not only accurate modeling but also cognitive and relational engagement between patients and care teams.

In “*User-centred Digital Solution for Dentofacial Deformities Management*,” Abbate et al. present a blueprint persona methodology for co-designing DT systems. By involving patients, clinicians, and engineers in early development, the authors demonstrate how user-centered design can bridge technical sophistication with empathy and usability, enhancing adoption and trust in digital health tools.

Equity and inclusion are central to Weinberger et al.'s “*Gender-Sensitive Digital Patient Twins: Towards Inclusive and Equitable Precision Medicine*”. The authors highlight the risks of bias in DT models trained on non-diverse datasets and call for gender-sensitive and intersectional design practices. They propose governance frameworks aligned with the EU AI Act and WHO ethics principles to ensure that digital twins promote fairness and clinical accuracy across populations.

At the population level, El-Warrak et al.'s “*Simulation-based Assessment of Digital Twin Systems for Immunisation*” introduces TwinVax, a DT platform for managing immunization logistics in primary care. Through UML-based system modeling and SimPy simulation, the study shows how DTs can monitor vaccine storage, reduce waste, and improve preventive care—demonstrating the potential of DTs to enhance public health resilience.

Implementation challenges are examined in “*Assessing Digital Twin Implementation Challenges in Healthcare Using the CFIR 2.0 Framework*” by Xames et al., who apply an implementation science approach to identify barriers across organizational, technological, and behavioral domains. Their findings highlight the need for hybrid frameworks that integrate sociotechnical and computational perspectives to ensure effective DT deployment.

A real-world interoperability example, described by Fernandez et al., comes from Brazil's national health data federation, where municipal and federal health infrastructures have been integrated using HL7-FHIR and OpenEHR standards. This initiative demonstrates how coordinated governance and standards-based architectures can overcome data fragmentation and support scalable, person-centered digital ecosystems.

Finally, a multi-centered study by Ibrahim et al. from South Ethiopia explores healthcare professionals' readiness for telemedicine adoption. With 68.8% of respondents expressing readiness, the study identifies knowledge, gender, attitude, and infrastructure as key factors that influence adoption. These insights underscore that human capacity and motivation are as critical as technology in enabling person-centric digital transformation.

## 3 Emerging trajectories and future directions

Across these contributions, three converging trajectories define the emerging landscape of digital twin implementation in healthcare:

- From data-centric to model-centric healthcare. DTs embody a paradigm shift toward dynamic, model-based reasoning that continuously integrates new data to refine clinical understanding and predict outcomes.
- From technological innovation to ethical and inclusive design. The evolution of DTs reflects an increasing focus on inclusivity, fairness, and participatory design, ensuring that these technologies serve diverse populations and foster equitable care.
- From isolated prototypes to interoperable ecosystems. Real-world impact requires integration across institutions and systems. Open standards, semantic interoperability, and shared governance are key to scaling DTs sustainably and securely.

Together, these directions signal the rise of digital twins as dialogic entities, mediating between patients, clinicians, and data systems to enable transparent, adaptive, and trustworthy healthcare.

## 4 Concluding remarks

This Research Topic illustrates the transition of digital twins from conceptual innovation to operational reality. Collectively, the papers emphasize that successful DT implementation requires more than computational excellence: it demands ethical alignment, human-centered design, and systemic interoperability. As digital health continues to evolve, DTs offer a pathway toward a learning healthcare ecosystem, one that is predictive, preventive, participatory, and person-centric. This vision can only be realized through sustained interdisciplinary collaboration that transforms digital innovation into living infrastructures for fair, transparent, and adaptive care.

